# Selective Glucocorticoid Receptor (GR-II) Antagonist Reduces Body Weight Gain in Mice

**DOI:** 10.1155/2011/235389

**Published:** 2011-07-28

**Authors:** Tomoko Asagami, Joseph K. Belanoff, Junya Azuma, Christine M. Blasey, Robin D. Clark, Philip S. Tsao

**Affiliations:** ^1^Department of Cardiovascular Medicine, Stanford University, Stanford, CA 94305, USA; ^2^Department of Psychiatry and Behavioral Sciences, Stanford University, Stanford, CA 94305, USA; ^3^Corcept Therapeutics, Menlo Park, CA 94025, USA

## Abstract

Previous research has shown that mifepristone can prevent and reverse weight gain in animals and human subjects taking antipsychotic medications. This proof-of-concept study tested whether a more potent and selective glucocorticoid receptor antagonist could block dietary-induced weight gain and increase insulin sensitivity in mice. Ten-week-old, male, C57BL/6J mice were fed a diet containing 60% fat calories and water supplemented with 11% sucrose for 4 weeks. Groups (*n* = 8) received one of the following: CORT 108297 (80 mg/kg QD), CORT 108297 (40 mg/kg BID), mifepristone (30 mg/kg BID), rosiglitazone (10 mg/kg QD), or vehicle. Compared to mice receiving a high-fat, high-sugar diet plus vehicle, mice receiving a high-fat, high-sugar diet plus either mifepristone or CORT 108297 gained significantly less weight. At the end of the four week treatment period, mice receiving CORT 108297 40 mg/kg BID or CORT 108297 80 mg/kg QD also had significantly lower steady plasma glucose than mice receiving vehicle. However, steady state plasma glucose after treatment was not highly correlated with reduced weight gain, suggesting that the effect of the glucocorticoid receptor antagonist on insulin sensitivity may be independent of its mitigating effect on weight gain.

## 1. Introduction

The role of insulin resistance in dietary-induced obesity is of increasing concern, as the prevalence of both obesity and type 2 diabetes approaches 1 in 3 in the United States population [1]. Mortality, morbidity, and health care costs mandate increased scientific effort towards discovery of underlying mechanisms of these illnesses in a quest to develop maximally effective therapies. 

The co-occurrence of excessive glucocorticoid activity and metabolic problems has long been noted [2]. In 2000, Chrousos formally hypothesized that stress-related hypercortisolism and glucocorticoid hypersensitivity are involved in the pathogenesis of the metabolic syndrome and obesity [3]. The potential intermediary role of cortisol in the connection between insulin sensitivity and weight gain can be readily observed from the extreme case of Cushing's syndrome. The defining characteristic of this syndrome is chronic elevation of circulating glucocorticoids; the hallmark symptoms of Cushing's syndrome are progressive truncal obesity and insulin resistance due to chronically elevated glucocorticoid levels [4]. Endogenous Cushing's syndrome is caused by corticotropin (ACTH)-producing pituitary tumors (Cushing's disease), by ectopic ACTH secretion from a nonpituitary tumor, and by cortisol secretion by an adrenal adenoma or carcinoma. The insulin resistance seen in Cushing's syndrome causes its major symptoms (obesity, glucose intolerance, hypertension, and dyslipidemia); it is thought that a similar mechanism is responsible for the metabolic syndrome [4, 5], although patients who have the metabolic syndrome by definition do not have ACTH or cortisol producing tumors. Pharmacological reduction of glucocorticoid activity can be achieved via several mechanisms of action, including neuromodulatory compounds which reduce corticotropin (ACTH) release from pituitary tumors, steroidogenesis inhibitors which reduce cortisol levels by adrenolytic activity and/or direct enzymatic inhibition, and finally glucocorticoid receptor (GR) antagonists which block cortisol action at its receptor [6, 7]. 

Manipulation of cortisol levels has been shown to alter insulin sensitivity [2, 8]. In vivo experiments have shown that glucocorticoids impair insulin-dependent glucose uptake in the periphery and enhance gluconeogenesis in the liver [9, 10]. Nieman et al. [11] reported the first case study demonstrating that mifepristone, a glucocorticoid antagonist, effectively corrected body weight gain and carbohydrate metabolism changes in Cushing's syndrome. Other investigational studies of Cushing's syndrome have shown that mifepristone exerts strong antiglucocorticoid effects and leads to rapid clinical improvement with acceptable side effects [12–16]. More recently, mifepristone has been reported to successfully ameliorate obesity and metabolic perturbations caused by antipsychotic medications in healthy humans [17, 18]. 

The purpose of this proof-of-concept study was to determine whether a newly identified, selective glucocorticoid receptor antagonist, CORT 108297, could block dietary-induced weight gain and increase insulin sensitivity in mice. Unlike mifepristone, CORT 108297 has no activity at the progesterone receptor. CORT 108297 was previously shown to attenuate weight gain induced by the antipsychotic medication olanzapine [19]. 

## 2. Materials and Methods

### 2.1. Animals and Diets

Forty (*N* = 40) ten-week-old, male, C57BL/6J mice were fed ad libitum a diet containing 60% fat calories (D12492, Research Diets Inc.) and water supplemented with 11% sucrose (decarbonated Sprite) for 4 weeks. In addition, they received one of the following five treatments: CORT 108297 (80 mg/kg QD), CORT 108297 (40 mg/kg BID), mifepristone (30 mg/kg BID), rosiglitazone, an oral glycemic medication (10 mg/kg QD), or vehicle (10% DMSO in 0.5% CMC). CORT 108297 is a selective glucocorticoid receptor antagonist and has a GR binding and functional profile similar to mifepristone with sub-nanomolar affinity for human GR and less than 10 nM GR functional activity in a luciferase-based reporter gene assay. Unlike mifepristone, it has no activity at the progesterone (PR) receptor [20]. An additional control group (*n* = 8) was fed a standard chow diet and tap water and did not receive any treatment.

### 2.2. Outcome Measures

The primary outcomes, body weight and insulin sensitivity, were measured weekly. Insulin sensitivity was measured by deriving steady state glucose values from the insulin suppression test (IST). Mice were fasted for 4 hours (from 8 AM to noon) prior to the IST. A combination of 300 *μ*L of saline with insulin (1 U/kg), glucose (1.3 g/kg), and somatostatin (0.13 mg/kg) was injected intraperitoneally. Blood glucose values were obtained from tail vein blood using a commercially available glucometer. Measurements were taken at 0, 60, 70, and 80 minutes post-injection. All procedures conformed to the Guides for Care and Use of Laboratory Animals of the National Institutes of Health and were approved by the Animal Subjects committee of Stanford University.

### 2.3. Statistical Analysis

For comparing change in weight across treatment groups, a multivariate analysis of variance model (MANOVA) was used with treatment group as a fixed factor. The primary analyses of interest defined *a priori* were the contrasts between the vehicle group (high-fat/high-sugar diet with no treatment) versus each active treatment group (i.e., CORT 108297 40 BID, CORT 108297 80 QD, mifepristone, and rosiglitazone). Analyses of steady state plasma glucose were conducted using nonparametric Mann-Whitney tests normalized to the *z* distribution. Associations between weight and steady state plasma glucose levels were tested using Pearson *r*.

## 3. Results

### 3.1. Body Weight

 There were statistically significant group differences in body weight gain (MANOVA *F* = 8.8, df = 5,48; *P* < .0001). The high-fat and high-sucrose diet induced significant weight gain: mice receiving the high-fat and high-sucrose diet but not treatment (i.e., the vehicle group) gained an average of 6 grams across the 4-week study (m = 6.3, sd = 1.7), whereas mice fed standard chow gained an average of 2 grams (M = 2.1, SD =  .9; *P* < .0001). Mice receiving either mifepristone or CORT 108297 in conjunction with the high-fat/ high-sucrose diet exhibited significantly less weight gain than mice receiving vehicle (mifepristone 30 mg/kg BID versus vehicle: *P* < .01; CORT 108297—80 mg/kg QD versus vehicle: *P* < .0001; CORT 108297—40 mg/kg BID versus vehicle: *P* < .0001). 

### 3.2. Steady State Plasma Glucose

 After 4 weeks, there were statistically significant differences between treatment groups in steady state plasma glucose. Compared to the group receiving a high-fat diet plus vehicle (M = 221, SD = 40), mice receiving CORT 108297 80 mg/kg QD (M = 196, SD = 28; Mann-Whitney *P* < .05) and mice receiving CORT 108297 40 mg/kg BID (M = 185, SD = 41; Mann-Whitney *P* < .05) had significantly lower steady state plasma glucose. Steady state plasma glucose was also significantly lower in the mice treated with mifepristone (M = 193, SD = 32; Mann-Whitney *P* < .05) or rosiglitazone (M = 161, SD = 38; Mann-Whitney *P* < .05). Mean SSPG and standard errors for each group are shown in Figure 2. 

#### 3.2.1. Association between Body Weight and Steady State Plasma Glucose

 Given that statistically significant mitigation of weight gain and steady state plasma glucose were both observed, a significant linear correlation was expected between weight change and plasma glucose levels. However, correlation analyses indicated no significant association between body weight gain and steady state plasma glucose (*r* = +.10, *P* = .98). Animals that exhibited less weight gain were no more likely to have lower plasma glucose values than animals that exhibited greater weight gain. 

## 4. Discussion

Significant body weight gain in mice was induced in a short period of time using a high-fat and high-sucrose diet. Mice receiving treatment with CORT 108297 in conjunction with this diet had significantly less body weight gain than vehicle-treated mice, suggesting that this selective glucocorticoid antagonist is potentially capable of mitigating dietary-induced weight gain. Insulin resistance was also increased due to the high-fat and high-sucrose diet. Mice receiving CORT 108297 had significantly less elevation in steady state glucose values than mice that received vehicle. 

In the current study, the effects of CORT 108297 on weight gain were comparable with the observed effects of mifepristone. The observed attenuation of weight gain is consistent with findings from previous animal studies and human clinical trials on mifepristone [17, 18, 21]. Two separate animal studies revealed that mifepristone [21] and CORT 108297 [19] attenuated medication-induced weight gain caused by ingestion of olanzapine, a medication consistently associated with obesity and metabolic problems. In humans, two randomized clinical trials showed that mifepristone significantly inhibited the body weight gain and metabolic disturbances caused by olanzapine and risperidone [17, 18]. Both mifepristone and CORT 108297 block activity at the glucocorticoid receptor, whereas CORT 108297, unlike mifepristone, does not block progesterone receptor activity. This implicates the blockade of glucocorticoid pathway—rather than the progesterone pathway—as the potential mechanism explaining mifepristone's previously observed effects on weight gain. 

 Whereas the prior literature demonstrated the role of glucocorticoid receptor antagonists for mitigating weight gain induced by antipsychotic medication usage, the current animal study implies that CORT 108297 can potentially suppress the weight gain induced by diet and, further, that it may increase insulin sensitivity. The concomitant effects of CORT 108297 on weight gain and steady state plasma glucose after four weeks of treatment in the current study must be viewed in the context of the nonsignificant correlation between the two variables. This result raises the possibility that the effects of CORT 108297 on the two outcomes are “independent” effects; that is, the attenuation of insulin sensitivity may not be merely a consequence of the compound's mitigating effect on weight gain. 

**Figure 1:**
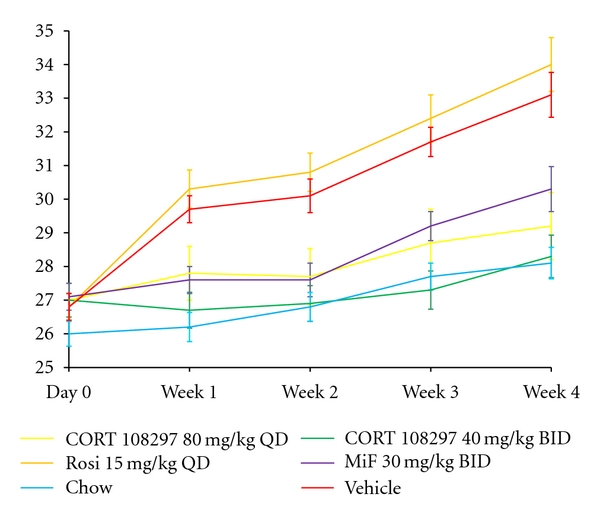
Mean body weight by treatment group. The mean body weights (grams) for the six groups of mice across the four week study. Vertical bars represent standard errors.

**Figure 2:**
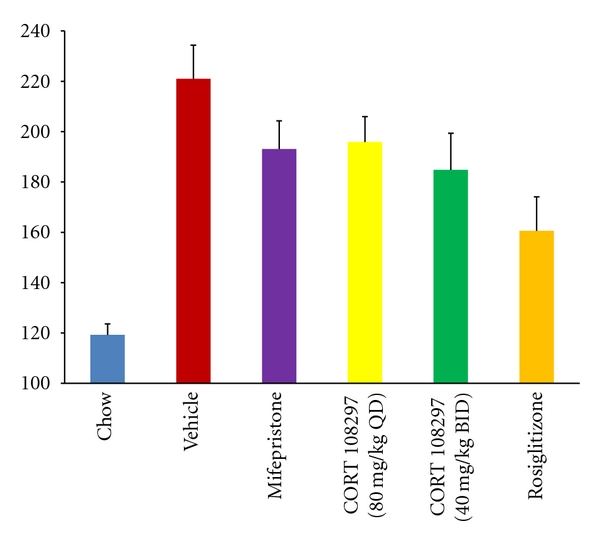
Steady state plasma glucose after 4 weeks. Height of bar represents the mean steady state plasma glucose value at the end of the four-week study. Thin bars indicate standard error.

Interestingly, mice receiving 40 mg/kg of CORT 108297 administered twice per day (i.e., total daily dose of 80 mg/kg) had less weight gain and lower plasma glucose compared with mice receiving 80 mg/kg once daily (see Figures 1 and 2). While not statistically significant, the directionality of this observation may reflect the relatively short half-life of CORT 108297 in rodents. 

 Inferences from this study are limited due to the proof-of-concept design which was not statistically powered to evaluate a large number of outcome variables. For example, this study did not collect data on food intake. Nonetheless, the effect sizes observed on the two outcomes that were measured, weight and insulin resistance, indicate that this line of research should be continued. Although larger sufficiently powered studies are needed, the lack of statistical association between weight gain and plasma glucose suggests that the effects of CORT 108297 on insulin sensitivity may occur independently of the GR antagonist's effects on weight. More comprehensive studies, which evaluate food intake and other metabolic measures, are warranted and could provide information about the underlying mechanisms involved in the relationship between glucocorticoids, weight gain, and insulin resistance. 

If the current findings are replicated and extended, CORT 108297 would offer an advantage over mifepristone given its lack of activity at the progesterone receptor. CORT 108297 is currently in Phase 2 testing.

##  Authorship Contributions

J. K. Belanoff and P. S. Tsao participated in research design. T. Asagami and J. Azuma conducted experiments. R. D. Clark contributed new reagents and analytic tool. C. M. Blasey and T. Asagami performed data analysis. T. Asagami, C. M. Blasey, and J. K. Belanoff wrote the manuscript.
